# Peritoneal transport status and first episode of peritonitis: a large cohort study

**DOI:** 10.1080/0886022X.2021.1949350

**Published:** 2021-07-07

**Authors:** Jing Hu, Hao Zhang, Bin Yi

**Affiliations:** Department of Nephrology, The Third Xiangya Hospital, Central South University, Changsha, China

**Keywords:** Peritonitis, technique failure, overall mortality, peritoneal transport status, risk factors

## Abstract

**Background:**

Peritonitis is one of the most serious complications of peritoneal dialysis (PD). This study aimed to explore the relationship between peritoneal transport status and the first episode of peritonitis, as well as the prognosis of patients undergoing continuous ambulatory peritoneal dialysis (CAPD).

**Method:**

A retrospective cohort study was conducted, analyzing data of CAPD patients from 1st January 2009, to 31st December 2017. Baseline data within 3 months after PD catheter placement was recorded. Cox multivariate regression analysis was performed to determine the risk factors for the first episode of peritonitis, technique failure and overall mortality.

**Results:**

A total of 591 patients were included in our analysis, with a mean follow-up visit of 49 months (range: 27–75months). There were 174 (29.4%) patients who had experienced at least one episode of peritonitis. Multivariate Cox regression analysis revealed that a higher peritoneal transport status (high and high-average) (HR 1.872, 95%CI 1.349–2.599, *p* = 0.006) and hypoalbuminemia (HR 0.932,95% CI 0.896, 0.969, *p* = 0.004) were independent risk factors for the occurrence of the first episode of peritonitis. In addition, factors including gender (male) (HR 1.409, 95%CI 1.103, 1.800, *p* = 0.010), low serum albumin (HR 0.965, 95%CI 0.938, 0.993, *p* = 0.015) and the place of residence (rural) (HR 1.324, 95%CI 1.037, 1.691, *p* = 0.024) were independent predictors of technique failure. Furthermore, low serum albumin levels (HR 0.938, 95%CI 0.895, 0.984, *p* = 0.008) and age (>65years) (HR 1.059, 95%CI 1.042, 1.076, *p* < 0.001) were significantly associated with the risk of overall mortality of PD patients.

**Conclusions:**

Baseline hypoalbuminemia and a higher peritoneal transport status are risk factors for the first episode of peritonitis. Factors including male gender, hypoalbuminemia, and residing in rural areas are associated with technique failure, while hypoalbuminemia and age (>65years) are predictors of the overall mortality in PD patients. Nevertheless, the peritoneal transport status does not predict technique failure or overall mortality of PD patients.

## Introduction

Peritonitis is one of the most serious complications of peritoneal dialysis (PD). The global rate for peritonitis across different peritoneal dialysis centers is approximately 0.06–1.16 per patient-year [1]. It is the main cause of technique failure of PD, resulting in catheter removal in 10%–88% (22% overall) [2] of patients and increased hospitalization rate and mortality [3–5]. Many risk factors have been implicated in the development of peritonitis, with several studies demonstrating that gender [6], age [7], ethnicity [8], end-stage kidney disease (ESKD) [9] are non-modifying risk factors, while obesity [10], smoking [11], residing far from the PD center [12], and albuminemia [13] are modifying risk factors.

Peritoneal transport status refers to the rate of peritoneal solute transport. It is determined by the peritoneal equilibration test (PET) and is an important basis for peritoneal dialysis prescription. Higher peritoneal transport is often associated with less ultrafiltration and high albumin loss. Studies have shown that the peritoneal transport status is closely related to the mortality of PD patients [14,15], but few studies have explored the relationship between the peritoneal transport status and the occurrence of peritonitis episodes [16]. Limited studies have concluded that the type of peritoneal transport does not predict the occurrence of peritonitis, while others have debated over the accuracy of this conclusion [17]. Therefore, we conducted a retrospective cohort study to investigate the relationship between baseline indicators, including peritoneal transport status and the first episode of peritonitis, as well as the long-term prognosis in CAPD patients.

## Materials and methods

In this study, only patients diagnosed with ESKD who had undergone catheter placement and attended regular follow-ups in our PD center from 1st January 2009 to 31st December 2017 were included. This study was approved by the Third Xiangya Hospital of Central South University (2019-S444). The exclusion criteria were patients under the age of 18 years old, those who had undergone catheter placement in other hospitals, patients who were prescribed PD after the failure of hemodialysis or renal transplantation, those who had a follow-up time shorter than 3 months, and patients with incomplete baseline clinical data.

The data collected included patient demographics [gender, age, place of residence (city/rural)], primary disease of ESKD, body mass index (BMI), baseline biochemical indicators (between 1 to 3 months after the initiation of peritoneal dialysis), white blood cells (WBC) count and hemoglobin count (g/L), the levels of serum albumin (g/L), potassium, calcium, phosphorus, blood urea nitrogen (BUN), creatinine (μmol/L), whole parathyroid hormone (iPTH), total triglyceride (TG), total cholesterol (CHO) and low-density lipoprotein (LDL). The PD adequacy index [kidney and peritoneal Kt/V (urea clearance index) urea] and peritoneal equilibration test (PET) results [dialysate-to-plasma ratio of creatinine (D/Pcr)] were also recorded. Patient follow-up data were collected until mortality occurred or until 1st September 2018. The dates of the first episode of peritonitis and the occurrences of PD technique failure were recorded.

Technique failure was defined as the event of permanent transfer from PD to hemodialysis. Overall survival was defined as the time from the start of PD to the time of death. The diagnostic criteria for peritonitis were adopted from the International Peritoneal Dialysis Association (ISPD) guidelines in the year 2000 [18]. For the diagnosis of PD-related peritonitis, two or more of the following must be met: (1) presence of clinical characteristics of peritonitis, such as abdominal pain and/or cloudy dialysis solution, (2) dialysate white blood cell count at a value of >100/μL or >0.1 × 109/L (with a dwell time of ≥2 h), polymorphonuclear cells >50%, (3) positive bacterial culture from the dialysate.

To determine the peritoneal transport status, a 4 h PET, as described by Twardowski et al. [19] was performed on each patient in the first 1–3 months after CAPD. Briefly, dialysate samples were taken at 0, 2 and 4 h of dwell, and blood samples were obtained at 2 h. Ratios of D:P creatinine were calculated using dialysate (D) creatinine concentrations at 4 h from the start of PET divided by the plasma (P) creatinine concentrations (4 h D:P cr). For statistical analyses, peritoneal transport status was categorized according to the 4 h D:P cr as below: low (<0.50); low-average (0.50–0.64); high-average (0.65–0.80); high (>0.81). Patients were divided into a lower peritoneal transport group (including low and low-average transporters) and a higher peritoneal transport group (including high-average and high peritoneal transporters).

Residual renal function was indicated by residual glomerular filtration rate (RGFR, ml/min/1.73 m^2^), which was calculated as the mean of creatinine and urea clearance, normalized to body size by body surface area.

### Statistical analysis

SPSS 21.0 software was used for statistical analysis. Data were presented as means and standard deviations or medians and interquartile range for continuous variables, and number (percentages) for categorical variables. Differences between the two groups were evaluated by Student’s t-test, Mann–Whitney test, or the Chi-square according to the types of the data. Differences in baseline characteristics of patients due to different transport statuses were evaluated by ANOVA. The Kaplan-Meier method was used to determine the survival time, the technique survival time, and the overall survival time of patients. The Log-Rank test was used to evaluate the difference in the survival rate. Univariate and multivariate Cox proportional hazard regression analyses were performed to evaluate the risk factors for the first episode of peritonitis, technique failure and overall mortality in PD patients. A P-value of <0.05 was considered statistically significant.

## Results

**Figure 1.**
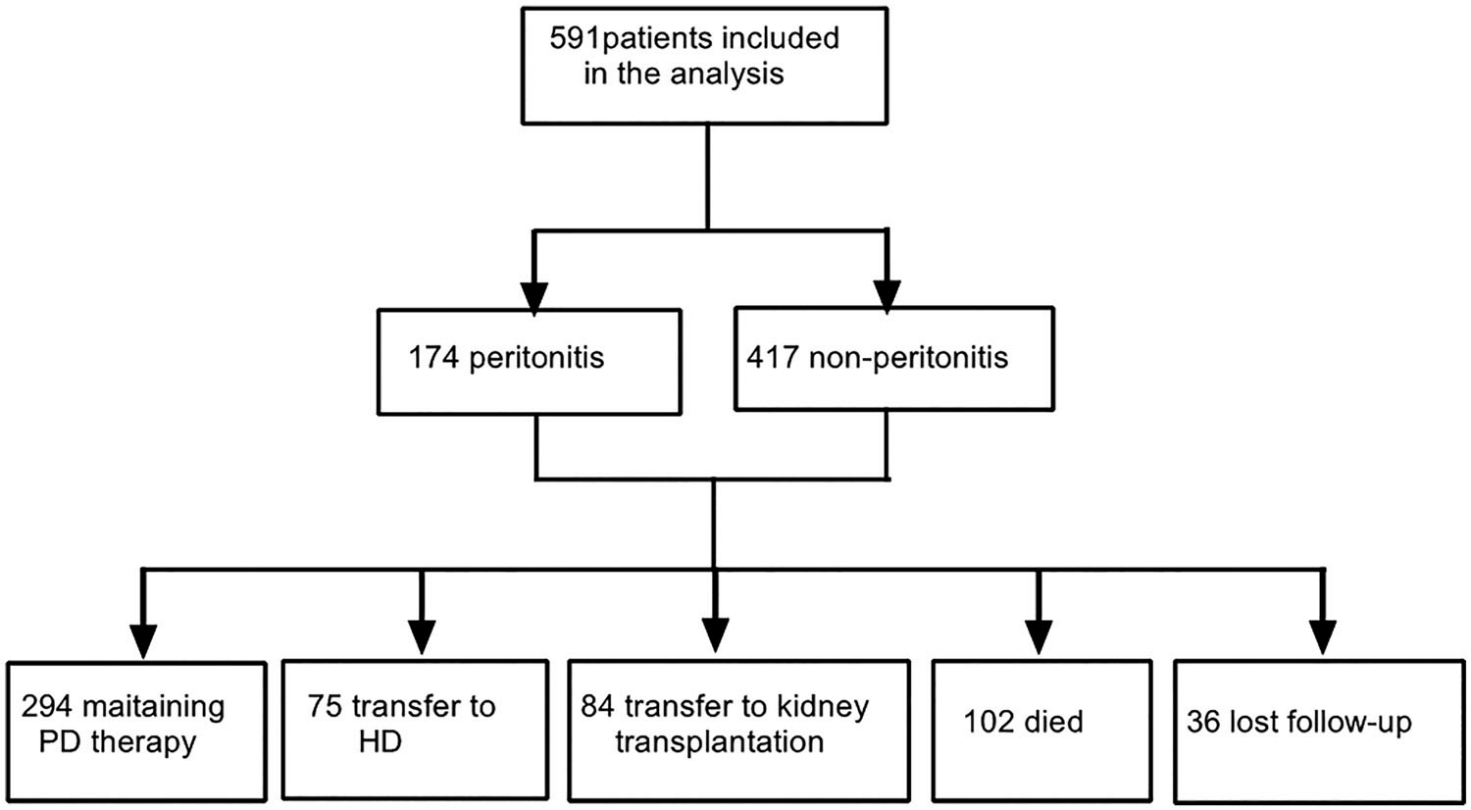
Screening process for enrolled patients.

A total of 591 patients were included in this study . Of these, 337 were male (57.0%), the mean age was 46.88 ± 13.47 years old when CAPD was started, and the mean follow-up time was 49 months (range: 27–75 months). All patients underwent an open insertion of a peritoneal dialysis catheter. The catheters were straight tubes made of double polyester. Calcium-containing peritoneal dialysis fluid (Baxter Healthcare Corporation, Deerfield, IL, USA) was used in all patients. The most common primary cause of ESKD was chronic glomerulonephritis (76.8%), followed by diabetic nephropathy (8.3%), hypertensive nephrosclerosis (4.9%) and obstructive nephropathy (6.1%) .During the follow-up visits, 75 patients (15.3%) were transferred from PD to hemodialysis, 84 patients (14.2%) underwent renal transplantation, 102 patients (17.3%) passed away, and 294 patients (49.7%) continued PD. There were 36 patients (6.1%) who did not attend follow-up visits. (Figure 1). The reasons for withdraw from peritoneal dialysis are shown in Table 1.

**Table 1.** Reasons for withdrawal from PD therapy.

Reasons	Data (;*n*,%)
Transfer to HD	75
Peritonitis	48 (64%)
Dialysis incomplete	12 (16%)
Ultrafiltration failure	10 (13.33%)
Psychosocial reasons	5 (6.67%)
Death	102
Cardiovascular diseases	34 (33.33%)
Cerebrovascular diseases	30 (29.41%)
Dialysis failure	13 (12.75%)
Alimentary tract hemorrhage	8 (7.84%)
Infection	6 (5.88%)
Multiple organ failure	5 (4.9%)
Others	6 (5.88%)

PD: peritoneal dialysis; HD: hemodialysis.

### Episodes of peritonitis

During the follow-up visits, 174 patients (29.4%) experienced at least one episode of peritonitis, with a total of 261 episodes. Of these, 118 patients had one episode of peritonitis, 37 patients had 2 episodes, 10 patients had three episodes, 6 patients had 4 episodes and 3 patients had 5 times episodes. The rate of peritonitis was 0.17 per patient-year (95% CI 0.15–0.19).

In this study, relapsing peritonitis was recorded as a single episode of peritonitis. Recurrent and repeat peritonitis was counted as another episode of peritonitis. From 2009 to September 2017, the yearly incidence of peritonitis in our center was 0.056 per patient-year (year 2009), 0.102 per patient-year (year 2010), 0.223 per patient-year (year 2011), 0.175 per patient-year (year 2012), 0.132 per patient-year (year 2013), 0.156 per patient-year (year 2014), 0.176 per patient-year (year 2015), 0.16 per patient-year (year 2016) and 0.145 per patient-year (year 2017).

### The first episode of peritonitis

From 0–12 months, 12–24 months and >24 months, the numbers of first episodes of peritonitis observed were 58 (33.3%), 41 (23.6%) and 75 (43.1%), respectively. Pathogens that were isolated during the first episode of peritonitis were: gram-positive bacteria (*n* = 61, 35.1%), gram-negative bacteria (*n* = 29, 16.7%), and fungi (*n* = 9, 5.2%). Among the gram-positive bacteria, the most frequently isolated strain was Coagulase-negative *staphylococci* (20.7%), followed by *Streptococcus* (6.3%), *Staphylococcus aureus* (5.7%), and *Enterococcus* (1.7%). Of the gram-negative bacteria, *Escherichia coli* was predominant and isolated in 7.5% of the cases, followed by *Acinetobacter bowman* (2.3%), *Klebsiella* (1.7%), and *Enterobacter cloacae* (1.2%). Following the first episode of peritonitis, the majority of patients (*n* = 147, 84.5%) recovered, while 25 patients (14.4%) were transferred from PD to hemodialysis, and 2 patients (1.1%) died. The outcomes of the first episode of peritonitis associated with different organisms were outlined in Table 2.

**Table 2.** Outcome of the different organisms infection for the first episode of peritonitis (*n* = 174).

Causative organisms	Number of cases (*n*)	Cure (*n*, %)	Transfer to HD (*n*, %)	Death (*n*, %)
Gram-positive organism	61	58 (95.1%)	3 (4.9%)	
Gram-negative organisms	29	23 (79.3%)	5 (17.2%)	1 (3.5%)
Fungi	9	4 (44.4%)	5 (55.6%)	
Multiple organisms	3	1 (33.3%)	1 (33.3%)	1 (33.3%)
No microbiological information	72	61 (84.7%)	11 (15.3%)	

HD: hemodialysis.

### Baseline data

Patients were categorized into the peritonitis group and the non-peritonitis group (Table 3). A significant difference was observed in the serum albumin levels and peritoneal transport types between the two groups. Patients in the peritonitis group had a lower level of serum albumin when compared with the non-peritonitis group.

**Table 3.** Baseline characteristics of patients.

Variable	Peritonitis (*n* = 174)	Nonperitonitis (*n* = 417)	*p*
Mean age (years)	49.86 ± 13.47	47.84 ± 12.972	0.051
Gender (male/female)	157/134 (1.17)	342/252 (1.35)	0.968
Diabetes mellitus (*n*, %)	25,14.3%	52,12.4%	0.681
Residence (city/rural)	115/176	283/311	0.232
BMI (kg/m^2^)	22.04 ± 3.28	22.18 ± 3.23	0.607
Hb (130–175g//L)	75.31 ± 16.71	75.83 ± 17.1	0.715
Serum albumin (40.0–55.0g//L)	32.85 ± 4.35	34.66 ± 4.44	<0.001
LDL-C (<2.59mmol/L)	2.16 ± 0.78	2.21 ± 0.79	0.452
TG (<1.7mmol/L)	1.35 ± 0.86	1.35 ± 0.83	0.938
CH (<4.41mmol/L)	4.09 ± 1.11	4.15 ± 1.16	0.551
BUN (2.6–8.8mmol/L)	26.66 ± 9.97	27.79 ± 10.97	0.184
Scr (41–81µmol/L)	973.79 ± 386.20	1003.57 ± 413.36	0.356
Potassium (3.5–5.3mmol/L)	4.41 ± 0.78	4.51 ± 0.84	0.138
Calcium (2.2–2.7mmol/L)	1.95 ± 0.32	1.93 ± 0.28	0.331
Phosphorus (0.85–1.51mmol/L)	1.99 ± 0.72	2.00 ± 0.65	0.910
iPTH (12–88pg/ml)	327.59 ± 275.76	315.75 ± 233.60	0.660
RGFR (ml/min/1.73 m^2^)	4.12 ± 1.88	3.89 ± 1.23	0.451
KT/V	2.1 ± 0.23	2.08 ± 0.36	0.543
Peritoneal transport status			<0.001
Low (*n*)	38 (21.84%)	140 (33.57%)	
Low-average (*n*)	63 (36.21%)	176 (42.21%)	
High-average (*n*)	50 (28.73%)	90 (21.58%)	
High (*n*)	23 (13.22%)	11 (2.64%)	

BMI: body mass index; Hb: hemoglobin; LDL-C: low-density lipoprotein cholesterol; TG: triglyceride; CH: Total cholesterol; BUN: blood urea nitrogen; Scr: serum creatinine; iPTH: intactparathyroidhormone; RGFR: residual glomerular filtrationrate; Kt/V: urea clearance index.

When the baseline data of patients were compared based on the different types of peritoneal transport status (Table 4), a significant difference in the mean age, the proportion of patients diagnosed with diabetes mellitus, and levels of serum albumin were observed between the groups. Patients in the higher transport group were older and had the highest proportion of patients diagnosed with diabetes mellitus. Patients in the higher peritoneal transport group had lower levels of serum albumin (*p* < 0.05) and creatinine (*p* < 0.05) than the lower peritoneal transport group.

**Table 4.** Baseline characteristics of patients in different transport types.

Variable	Low (*n* = 178)	Low-average (*n* = 239)	High-average (*n* = 140)	High (*n* = 34)	*p*
Gender (male/female)	95/83 (1.14)	176/63 (2.74)	90/50 (1.8)	23/11 (2.09)	<0.001
Mean age (years)	44.84 ± 13.22	46.40 ± 13.01	49.20 ± 12.88	51.03 ± 12.72	0.006
Diabetes mellitus (*n*,%)	4,2.25%	17,7.11%	22,15.71%	6,17.65%	<0.001
BMI (kg/m^2^)	21.62 ± 3.1	22.19 ± 3.2	22.12 ± 3.1	21.38 ± 2.9	0.198
Hb (130–175g//L)	78.86 ± 17.86	75.13 ± 16.7	75.32 ± 17.31	78.44 ± 18.82	0.123
Serum albumin (40.0–55.0g//L)	35.19 ± 4.8	33.8 ± 4.3	33.89 ± 3.89	33.12 ± 4.6	0.004
LDL-C (<2.59mmol/L)	2.14 ± 0.75	2.22 ± 0.76	2.26 ± 0.85	2.18 ± 0.87	0.622
TG (<1.7mmol/L)	1.31 ± 0.85	1.42 ± 0.88	1.3 ± 0.88	1.40 ± 0.97	0.505
CH (<4.41mmol/L)	4.05 ± 1.28	4.18 ± 1.05	4.22 ± 1.17	4.29 ± 1.20	0.518
BUN (2.6–8.8mmol/L)	28.56 ± 11.7	28.14 ± 11.41	26.41 ± 10.16	26.78 ± 8.31	0.317
Scr (41–81µmol/L)	1032.42 ± 423.57	1018.35 ± 406.01	985.39 ± 426.03	961.18 ± 409.88	0.671
Potassium (3.5–5.3mmol/L)	4.43 ± 0.83	4.47 ± 0.83	4.55 ± 0.77	4.47 ± 0.98	0.594
Calcium (2.2–2.7mmol/L)	1.96 ± 0.27	1.96 ± 0.34	1.96 ± 0.31	1.91 ± 0.37	0.764
Phosphorus (0.85–1.51mmol/L)	1.98 ± 0.68	2.06 ± 0.68	1.94 ± 0.64	2.19 ± 0.80	0.234
iPTH (12–88pg/ml)	319.97 ± 190.12	300.05 ± 106.34	323.92 ± 112.43	242.13 ± 105.96	0.493
KT/V	1.94 ± 0.13	1.96 ± 0.21	2.12 ± 0.33	2.15 ± 0.23	0.078

BMI: body mass index; Hb: hemoglobin; LDL-C: low-density lipoprotein cholesterol; TG: triglyceride; CH: Total cholesterol; BUN: blood urea nitrogen; Scr: serum creatinine; iPTH: intactparathyroidhormone; Kt/V: urea clearance index.

### Influence of peritonitis on technique failure and overall mortality

**Figure 2.**
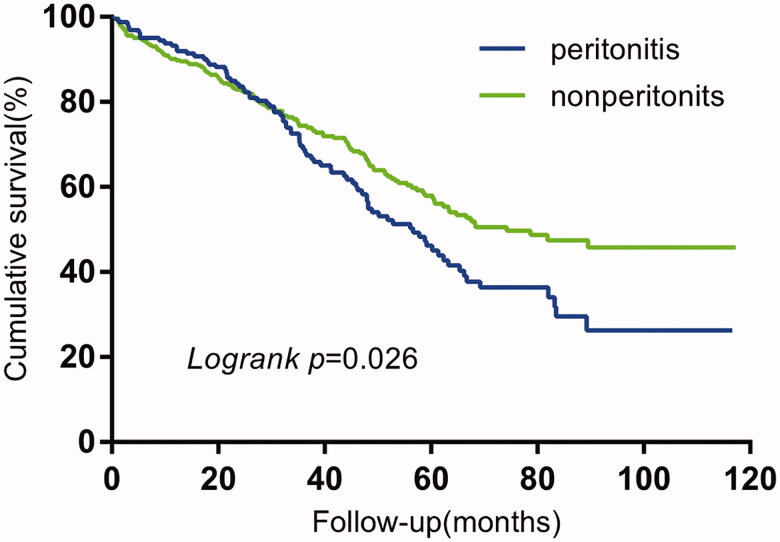
Kaplan-Meier curves of comparative technique survival by peritoneal dialysis patients of peritonitis VS nonperitonitis.

**Figure 3.**
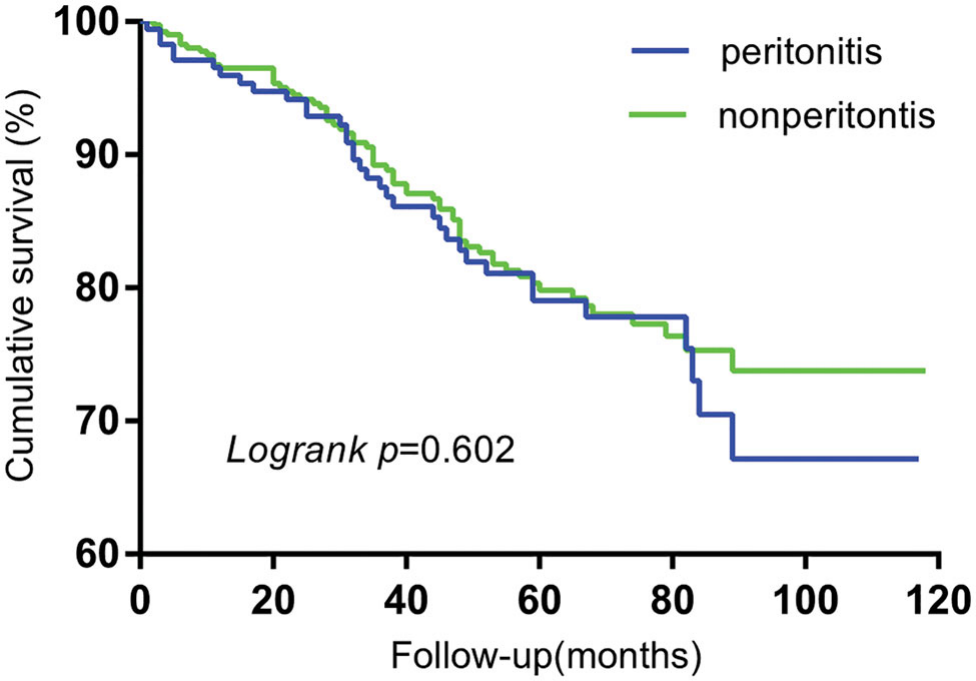
Kaplan-Meier curves of comparative overall survival by peritoneal dialysis patients of peritonitis VS nonperitonitis.

The technique survival (*p* = 0.026) in the peritonitis group was significantly shorter than that of the non-peritonitis group, while no significant difference (*p* = 0.602) was observed in the overall survival between the two groups (Figures 2 and 3).

### Risk factors for the first episode of peritonitis

Univariate Cox regression analysis showed that factors including patient’s age, the level of serum albumin, and the type of peritoneal transport were associated with the onset of the first episode of peritonitis. In the multivariate Cox proportional hazards model (Table 5), low levels of serum albumin (HR = 0.932, 95% CI 0.896–0.969, *p* = 0.004) and high peritoneal transport type (high and high-average) (HR = 1.872, 95% CI 1.349–2.599, *p* = 0.006) were independent risk factors for the first episode of peritonitis. The risk of peritonitis was increased by 6.8% for every 1 g/L decrease in the baseline serum albumin level.

**Table 5.** Risk factors of first peritonitis episode.

	Univariate Analysis			Multivariate Analysis		
Variable	HR	(95% CI)	*p*	HR	(95%CI)	*p*
Mean age(>65years)	1.014	1.002–1.026	0.020	1.041	0.595–1.822	0.888
Gender (male)	1.001	0.743–1.351	0.992			
Diabetes mellitus	1.624	1.108–2.591	0.260			
Residence (city vs. country)	0.890	0.675–1.205	0.452			
BMI (kg/m^2^)	0.982	0.935–1.032	0.470	0.981	0.929–1.037	0.505
Hb (130–175g//L)	0.916	0.991–1.008	1.000			
Serum albumin (40.0–55.0g//L)	0.922	0.890–0.955	<0.001	0.932	0.896–0.969	0.004
LDL-C(<2.59mmol/L)	0.895	0.726–1.104	0.299			
TG (<1.7mmol/L)	0.985	0.827–1.127	0.866			
CH (<4.41mmol/L)	0.967	0.843–1.110	0.638			
BUN (2.6–8.8mmol/L)	0.990	0.977–1.003	0.132			
Scr(41–81µmol/L)	1.000	0.999–1.000	0.322			
Potassium (3.5–5.3mmol/L)	0.913	0.722–1.155	0.448			
Calcium (2.2–2.7mmol/L)	1.240	0.746–2.061	0.406			
Phosphorus (0.85–1.51mmol/L)	1.015	0.890–1.214	0.457			
iPTH (12–88pg/ml)	0.999	0.998–1.001	0.263			
eGFR(ml/min/1.73 m^2^)	0.891	0.872–1.023	0.326			
KT/V	0.876	0.624–1.003	0.453			
Peritoneal transport status	1.765	1.306–2.386	<0.001	1.872	1.349–2.599	0.006
(H,HA vs.L,LA)						

BMI: body mass index; Hb: hemoglobin; LDL-C: low-density lipoprotein cholesterol TG: triglyceride; CH: total cholesterol; BUN: blood urea nitrogen; Scr: serum creatinine; iPTH: intactparathyroidhormone; RGFR: residual estimated glomerular filtrationrate; Kt/V: urea clearance index; H: high; HA: high-average; L:low; LA: low-average.

### Influence of peritoneal transport status on technique failure and overall mortality

From the Kaplan-Meier analyses, the type of peritoneal transport status was not associated with technique failure (*p* = 0.116) or overall mortality (*p* = 0.071).

### Risk factors for technique failure

Univariate Cox regression analysis demonstrated that factors including diabetes as the primary disease, gender, age, serum albumin levels, and place of residence were associated with technique failure. In the multivariate Cox regression analysis, male gender (HR = 1.409, 95% CI 1.103–1.800, *p* = 0.010), hypoalbuminemia (HR = 0.965, 95% CI 0.938–0.993, *p* = 0.015) and residing in rural areas (HR = 1.324, 95% CI 1.037–1.691, *p* = 0.024) were the independent risk factors for PD technique failure (Table 6).

**Table 6.** Risk factors of technique failure.

	Univariate analysis			Multivariate analysis		
Variable	HR	(95% CI)	*p*	HR (95% CI)		*p*
Mean age(>65years)	1.206	0.840–1.733	0.043	1.004	0.994–1.014	0.448
Gender (male)	1.500	1.583–1.983	0.002	1.409	1.103–1.800	0.010
Diabetes mellitus	1.410	0.976–2.036	0.060	1.098	0.742–1.627	0.639
Residence(rural)	1.396	1.113–1.755	0.004	1.342	1.037–1.691	0.024
BMI (kg/m2)	0.887	0.922–1.073	0.470			
Hb (130–175g//L)	0.993	0.978–1.007	0.327			
Serum albumin (40.0–55.0g//L)	0.994	0.940–0.992	0.010	0.965	0.938–0.968	0.015
LDL-C (<2.59mmol/L)	1.591	0.823–3.704	0.167			
TG (<1.7mmol/L)	0.952	0.663–1.367	0.789			
CH (<4.41mmol/L)	0.995	0.615–1.613	0.985			
BUN (2.6–8.8mmol/L)	0.993	0.960–1.208	0.696			
Scr(41–81µmol/L)	1.001	0.999–1.001	0.141			
Potassium (0.85–1.51mmol/L)	1.224	0.907–1.652	0.855			
Calcium (2.2–2.7mmol/L)	1.407	0.592–3.344	0.439			
Phosphorus(0.85–1.51mmol/L)	1.015	0.890–1.214	0.27			
iPTH (12–88pg/ml)	0.999	0.998–1.001	0.304			
RGFR(ml/min/1.73 m2)	0.991	0.925–1.063	0.809			
KT/V	0.876	0.624–1.003	0.453			
Peritoneal transport status	1.102	0.663–1.834	0.707			
(H,HA vs.L,LA)						

BMI: body mass index; Hb: hemoglobin; LDL-C: low-density lipoprotein cholesterol TG: triglyceride; CH: total cholesterol; BUN: blood urea nitrogen; Scr: serum creatinine; iPTH: intactparathyroidhormone; RGFR: residual glomerular filtrationrate; Kt/V: urea clearance index; H: high; HA: high-average; L: low; LA: low-average.

### Risk factors for overall mortality

Univariate Cox regression analysis revealed that factors including the age of the patient at catheter placement, serum albumin level, having diabetes as the primary disease and the place of residence were associated with the overall survival of the patients. In the multivariate Cox regression analysis, age >65years (HR = 1.059, 95% CI 1.042–1.076, *p* < 0.001) and hypoalbuminemia at the time of catheter placement (HR = 0.938, 95% CI 0.895–0.984, *p* = 0.008) were independent risk factors for overall mortality (Table 7).

**Table 7.** Risk factors of overall mortality.

	Univariate Analysis			Multivariate Analysis		
Variable	HR	(95% CI)	*p*	HR (95% CI)		*p*
Mean age (>65years)	3.186	1.002–1.026	<0.001	1.059	1.042–1.076	<0.001
Gender (male)	2.246	0.700–7.210	0.174			
Diabetes mellitus	2.791	1.694–4.599	<0.001	1.766	1.019–1.365	0.401
Residence (rural)	1.611	1.092–2.375	0.016	0.836	0.553–1.263	0.394
BMI (kg/m2)	0.923	0.774–1.100	0.369			
Hb (130–175g//L)	0.962	0.991–1.000	0.068			
Serum albumin (40.0–55.0g//L)	0.921	0.880–0.964	<0.001	0.938	0.895–0.984	0.008
LDL (<2.59mmol/L)	4.991	1.076–23.145	0.060			
TG (<1.7mmol/L)	1.508	0.403–2.781	0.908			
CH (<4.41mmol/L)	0.585	0.186–1.837	0.358			
BUN (2.6–8.8mmol/L)	0.984	0.916–1.057	0.653			
Scr(41–81µmol/L)	0.999	0.997–1.001	0.183			
Potassium (3.5–5.3mmol/L)	2.23	1.074–4.630	0.331			
Calcium (2.2–2.7mmol/L)	0.345	0.043–2.751	0.315			
Phosphorus (0.85–1.51mmol/L)	1.443	0.890–1.214	0.496			
iPTH (12–88pg/ml)	0.997	0.993–1.001	0.149			
RGFR (ml/min/1.73 m2)	0.985	0.857–1.133	0.837			
KT/V	0.876	0.624–1.003	0.453			
Peritoneal transport status	1.839	0.573–5.902	0.306			
(H,HA vs.L,LA)						

BMI: body mass index; Hb: hemoglobin; LDL-C: low-density lipoprotein cholesterol; TG: triglyceride; CH: total cholesterol; BUN: blood urea nitrogen; Scr: serum creatinine; iPTH: intactparathyroidhormone; RGFR: residual glomerular filtrationrate; Kt/V: urea clearance index; H: high; HA: high-average; L: low; LA: low-average.

## Discussion

This retrospective cohort study included a large sample size of PD patients revealed that low serum albumin levels and higher peritoneal transport status at baseline were independent risk factors for PD-associated first episode of peritonitis in CAPD patients, but the type of peritoneal transport was not associated with the technique failure or overall mortality. In addition, factors including male gender, hypoalbuminemia and residing in rural areas were the independent risk factors for the technique failure, while age >65 years and hypoalbuminemia at the time of peritoneal dialysis initiation were risk factors for the overall mortality of CAPD patients.

Serum albumin is typically used as an indicator of the nutritional status of patients, although hypoalbuminemia may also be associated with inflammation. Serum albumin <3.8 g/dL represents one of the clinical diagnostic criteria of protein energy wasting (PEW) in patients with chronic kidney disease (CKD) [20]. Our analysis demonstrated that a low level of baseline serum albumin was a significant risk factor for the first onset of PD-related peritonitis, which is consistent with previous reports. Numerous studies have associated a low level of serum albumin with the occurrence of PD-related peritonitis [21–23]. According to the Zhongshan Yiyi Research Group, the baseline hypoalbuminemia is an important predictor of peritonitis and a risk factor for the first episode of peritonitis [24], as it increases the risk of early-onset peritonitis by 75%. Other studies from China [25] have also shown that the risk of developing early-onset peritonitis increases by 5.0% for every 1 g/L reduction in the serum albumin level. Other studies from Hong Kong [23] and the United States [22] have also revealed a 67% and 74% increase, respectively, in the risk of peritonitis for every 10 g/L reduction in the baseline serum albumin levels. Consistently, our results showed that the risk of first-onset peritonitis increased by 6.8% for every 1 g/L reduction in the baseline serum albumin level. Hypoalbuminemia leading to an increased risk of peritonitis could be attributed to the albumin-related inhibition of apoptosis of peritoneal macrophages. The level of albumin is a major factor in the survival of peritoneal macrophages. In a laboratory experimental study, the bovine serum albumin has been shown to inhibit the apoptosis of murine peritoneal macrophage cells, subjected to survival factor withdrawal by both lipid-dependent and independent mechanisms [26]. Apoptosis inhibition is dependent on scavenging reactive oxygen species (ROS) and the release of lysophosphatidic acid, which can result in decreased abdominal defense and poor nutritional status. Additionally, poor nutrition has been shown to reduce both cellular and humoral immunity [27], leading to an increased risk of infection. Our results also showed that a low level of serum albumin was a risk factor for the occurrence of technique failure of PD, which might be related to the increase in the incidence of peritonitis. Unsurprisingly, the rate of technique failure in our study was higher in the peritonitis group than in the non-peritonitis group. These findings were consistent with previous studies [28–30], with peritonitis associated directly with up to 20% of technique failure of PD. Furthermore, several studies have shown that a low level of serum albumin is a strong predictor of mortality in PD patients [31–33] and increases mortality by 2–6% [5,22,34]. Consistently, our multivariate Cox regression analysis revealed that a low level of baseline serum albumin was an independent risk factor for the death of PD patients, with a 6.2% increase in mortality for every 1 g/L reduction in the serum albumin level.

A higher transport state of the peritoneum to small molecule solute is often accompanied by insufficient ultrafiltration and excessive protein loss, leading to extreme volume overload, hypoalbuminemia, malnutrition and poor prognosis of PD. Also, a meta-analysis [15] has shown that increased peritoneal transport is associated with an increased risk of mortality, and an increased tendency to develop technique failure. A study from China has also associated higher peritoneal transport with mortality in short-term PD patients [35]. However, the relationship between the peritoneal transport status and PD-related peritonitis remains unclear. Apart from the study by Ning et al. [36], several small-sample size studies [17] have demonstrated a correlation between peritoneal transport function and peritonitis. Inconsistencies in results from various studies may be attributed to the differences in sample size, follow-up time, and groupings of patients for analyses. In our study, higher peritoneal transport was a risk factor for the first-onset of peritonitis (HR = 1.872, 95%CI 1.349–2.599), which could be due to the followings: (1) high protein loss from peritoneum in patients with a higher transport status, resulting in hypoalbuminemia and malnutrition [37,38]. Our analysis showed a positive correlation between higher transport type and lower baseline serum albumin levels. An increase in protein loss in the dialysate may result in hypoproteinemia, lipid abnormalities, and decreased antioxidant capacity [39,40]; (2) low serum albumin and volume overload caused by high peritoneal transport are associated with chronic inflammation [41], leading to malnutrition-inflammation-atherosclerosis (MIA) syndrome, which is an independent risk factor for peritonitis [22]; (3) high peritoneal transport reduces ultrafiltration capacity, resulting in excessive volume overload, increased incidence of hypertension [42], increased tendency of hypertrophy of the left ventricular and ultimately increased likelihood of heart failure [43], which are all associated with the onset of peritonitis [44]; (4) in PD patients with high transport the dwell time is shortened to increase the ultrafiltration volume, leading to a decline in the number of macrophages and phagocytosis in dialysate, which significantly decreases the activity of opsonins such as IgG and C3, thereby adversely affecting the ability of peritoneal defense [45]; (5) our analysis showed a higher proportion of diabetic patients in the high transport group, with many studies demonstrating diabetes as one of the risk factors for the incidence of peritonitis [46,47]. Nevertheless, our results did not support the association between the peritoneal transport type and the technique failure or overall mortality of PD patients, which is consistent with previous studies.

Factors including the baseline hypoalbuminemia, gender (male) and place of residence (rural) have not been consistently shown to affect technique failure in PD patients. While some studies have shown that female patients tend to attend fewer follow-up sessions [48], others have observed no difference in the consistency of follow-up visits between genders [49]. These disparities may be attributed to the social and cultural differences in different PD centers. The rate of technique failure for patients living in the rural areas is higher than those living in the town areas, which could be due to rural patients being far from the PD center [12], do not consistently attend follow-up sessions, have more medical complications, have a relatively lower level of education [48,50] and lacking in knowledge of PD. Nevertheless, our study did not investigate the association between the level of patient education and the rate of technique failure in PD.

Our study revealed that the age at the time of peritoneal catheterization was significantly associated with the mortality of PD patients, with age >65 years being the independent risk factor for the overall mortality. The risk of mortality increases with advancing age, which is consistent with findings from other PD centers. The ANZDATA study from Australia and New Zealand has shown a higher risk of mortality in elderly dialysis patients, with 74% of the mortality observed in those of 65–84 years old [48]. This could be due to a higher possibility of complications occurring in elderly patients.

There were strengths and limitations to this study. Our analyses were based on a large patient sample size. Also, the time period of this study was long with numerous follow-up sessions with patients. However, our patients were all from a single center and therefore might be subjected to selection bias. Also, being a retrospective study, there could be other confounding factors or covariates that were not taken into consideration in our analyses.

## Conclusions

Baseline hypoalbuminemia and high peritoneal transport are significant risk factors for the first incidence of PD-related peritonitis. The type of peritoneal transport, however, is unrelated to the technique failure and overall mortality of PD patients. Factors including male gender, hypoalbuminemia and residing in rural areas are associated with technique failure, while older age (>65years) and hypoalbuminemia at the time of peritoneal catheterization are independent predictors of the overall mortality of PD patients. Our findings shed light on the high-risk group of PD patients with poor prognosis, which enables timely intervention such as strengthening nutritional intake and follow-up procedures. This may ultimately improve the outcomes of patients undergoing peritoneal dialysis.
